# Targeting SRC Family Kinases in Mesothelioma: Time to Upgrade

**DOI:** 10.3390/cancers12071866

**Published:** 2020-07-11

**Authors:** Paola Indovina, Iris Maria Forte, Francesca Pentimalli, Antonio Giordano

**Affiliations:** 1 Sbarro Institute for Cancer Research and Molecular Medicine, Center for Biotechnology, College of Science and Technology, Temple University, Philadelphia, PA 19122, USA; giordano@temple.edu; 2 Institute for High Performance Computing and Networking, National Research Council of Italy (ICAR-CNR), I-80131 Naples, Italy; 3 Cell Biology and Biotherapy Unit, Istituto Nazionale Tumori-IRCCS-Fondazione G. Pascale, I-80131 Naples, Italy; m.forte@istitutotumori.na.it; 4 Department of Medical Biotechnologies, University of Siena, I-53100 Siena, Italy

**Keywords:** SRC family kinases, malignant mesothelioma, receptor tyrosine kinases, p27, AKT, invasion, dasatinib, treatment resistance, predictive signature, drug combination

## Abstract

Malignant mesothelioma (MM) is a deadly tumor mainly caused by exposure to asbestos. Unfortunately, no current treatment is able to change significantly the natural history of the disease, which has a poor prognosis in the majority of patients. The non-receptor tyrosine kinase SRC and other SRC family kinase (SFK) members are frequently hyperactivated in many cancer types, including MM. Several works have indeed suggested that SFKs underlie MM cell proliferation, survival, motility, and invasion, overall affecting multiple oncogenic pathways. Consistently, SFK inhibitors effectively counteracted MM cancerous features at the preclinical level. Dasatinib, a multi-kinase inhibitor targeting SFKs, was also assessed in clinical trials either as second-line treatment for patients with unresectable MM or, more recently, as a neoadjuvant agent in patients with resectable MM. Here, we provide an overview of the molecular mechanisms implicating SFKs in MM progression and discuss possible strategies for a more successful clinical application of SFK inhibitors. Our aim is to stimulate discussion and further consideration of these agents in better designed preclinical and clinical studies to make the most of another class of powerful antitumoral drugs, which too often are lost in translation when applied to MM.

## 1. Introduction

Malignant mesothelioma (MM) is a very aggressive cancer of the serous membranes lining the body cavities: approximately 70–80% of MMs develop in the pleura, 20% in the peritoneum, and a minority affects the pericardium and tunica vaginalis [1]. The main risk factor for MM is exposure to asbestos [2,3], which is implicated in ~80% of cases [1]. Although the use of asbestos has been banned in many countries, MM burden is still substantial, with over 30,000 cases and over 25,000 deaths worldwide in 2018 [4]. MM incidence is expected to further rise because of the long-latency time between exposure and diagnosis [5]. Moreover, asbestos is still used in the developing countries and the use of other mineral fibers that are known to cause MM, such as erionite, is not strictly regulated [6].

MMs are classified into three main histologic subtypes: epithelioid, sarcomatoid, and biphasic, characterized, respectively, by epithelial cells, spindle-shaped cells, or both cell types [7,8]. Sarcomatoid tumors determine the poorest outcome [6,8], although all histotypes have a very poor prognosis. Patients with MM of the pleura have a median survival of around 1 year from diagnosis [9]. At present, there is indeed no known curative modality for this cancer type. Surgery is a possible option, but the high risk of perioperative morbidity and mortality and the lack of consistent data from studies comparing outcomes of surgical and non-surgical approaches render its role very controversial; thus, surgery might be appropriate only for carefully and highly selected patients, depending on their general medical status and the type and site of cancer [3,9,10]. Only one first-line treatment has been approved by the Food and Drug Administration since 2003: a combination of a platinum compound with an antifolate (often cisplatin + pemetrexed), which has unfortunately shown only limited effects [9,11]. No treatments are approved in the second-line setting, despite clinical evaluation of several possible therapeutic targets over the years [11].

Profound knowledge of MM molecular alterations and tumor heterogeneity is needed to identify new druggable vulnerabilities and develop successful “precision medicine” strategies [11,12,13]. Mutations/copy-number loss of tumor suppressor genes, such as cyclin-dependent kinase inhibitor 2A (*CDKN2A*), neurofibromin 2 (*NF2*), and BRCA1 associated protein 1 (*BAP1*), are the most frequent genetic alterations in MM [14,15,16]. In particular, the homozygous deletion of the *CDKN2A* locus causes functional inactivation of both p53 and RB1 tumor suppressor pathways [16]. Interestingly, this genomic deletion frequently involves also an adjacent metabolic gene (methylthioadenosine phosphorylase, *MTAP*), thus generating a targetable vulnerability, which can be exploited therapeutically against MM, as we have recently suggested [17]. *NF2* deficiency, reported in 40% of MM cases [18], leads to inactivation of the tumor-suppressive Hippo pathway [19] and activation of multiple oncogenic pathways, which promote cell proliferation, migration, and survival [20]. *BAP1* germline mutations have recently been discovered to predispose to MM [21]; moreover, *BAP1* resulted the most commonly mutated gene in MM, with somatic alterations occurring in over 60% of sporadic cases [22,23].

Although oncogenic activating mutations in crucial regulators of growth and survival signaling, such as receptor tyrosine kinases (RTKs), rarely occur in MM, these kinases and downstream pathways are frequently overexpressed and hyperactivated in this cancer [11,13,14,24]. In particular, deregulated RTKs in MM include epidermal growth factor receptor (EGFR), MET (also known as hepatocyte growth factor receptor, HGFR), platelet-derived growth factor receptor (PDGFR), insulin-like growth factor 1 receptor (IGF1R), fibroblast growth factor receptor 1 (FGFR1), colony-stimulating factor 1 receptor (CSF-1R), and vascular endothelial growth factor receptor (VEGFR); whereas downstream effectors commonly altered include the mitogen-activated protein kinase (MAPK)/ERK pathway (also known as RAS/RAF/MEK/ERK pathway) and phosphatidyl-inositol 3-kinase (PI3K)-AKT pathway [11,13,14,24].

In line with the activation of the abovementioned RTKs and downstream pathways, SRC and other SRC family kinases (SFKs), which are non-receptor tyrosine kinases cooperatively interacting with RTKs and transducing their signals to downstream effectors [25,26,27,28,29,30], are also frequently hyperactivated in MM [31,32]. SFKs represent appealing targets for cancer therapy because of their involvement in several key processes underlying tumor development and progression in many tumor types [25,27,28,29]. Different studies have implicated a role of SFKs in multiple pathways altered in MM (as discussed below), proposing them as actionable therapeutic targets.

Many small molecules inhibiting SFKs and multiple other kinases (hereafter called “SFK inhibitors”, for simplicity) have been developed [33,34,35,36,37,38,39,40,41,42,43,44], some of which have also been used in MM preclinical [45,46,47,48,49] and clinical studies [50,51] (Table 1).

In preclinical studies, SFK inhibitors proved antitumor activity in MM cells, both alone [32,49] and in combination with either the chemotherapeutic agents currently used for MM treatment [47,48,52,53] or other tyrosine kinases inhibitors [31]. However, despite these encouraging preclinical observations, SFK inhibition was not successful in early clinical trials in MM patients [50,51].

In this review, after briefly describing the SFK structure, mechanisms of activation, and functions, we discuss the studies exploring the role of SFKs in MM progression and treatment. We also consider the main hurdles for a successful translation of SFK inhibition in the clinical setting and possible future directions.

## 2. SFK Structure and Activation

*SRC* was first identified in 1976 as the cellular counterpart of the transforming gene of the avian Rous sarcoma virus, *v-src*, which was the first viral oncogene to be discovered [54]. Since its milestone discovery, other variants, including FYN, YES, BLK, FGR, HCK, LCK, YRK, LYN, FRK, have been found and collectively termed SFKs [27].

**Figure 1:**
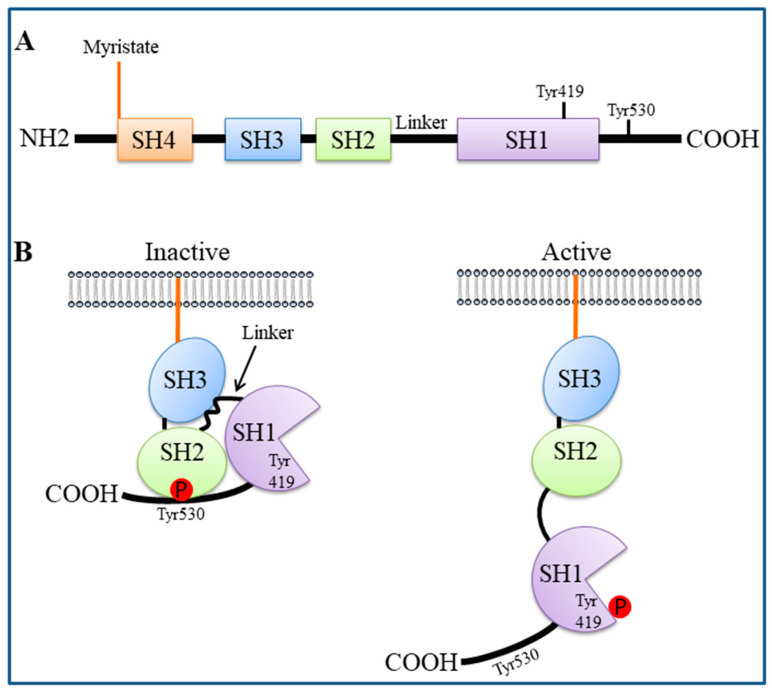
Structure and activation of SRC. (**A**) SRC is composed of four SRC homology (SH) domains: SH4 contains signals for myristoylation for membrane localization; SH3 and SH2 are protein binding regions; SH1 is the tyrosine kinase domain. A linker sequence lies between SH1 and SH2. (**B**) In its inactive form, SRC is phosphorylated in the C-terminal tail at Tyr530 and adopts a closed conformation: the phosphorylated C-terminus binds the SH2 domain and the linker region binds the SH3 domain. Dephosphorylation of Tyr530 causes a conformational change, resulting in autophosphorylation of the kinase domain at Tyr419 and full activation.

SFK members are composed of the following domains (from N- to C-terminus): the SRC homology (SH) 4 domain, which contains signals for myristoylation (in all SFKs) and palmitoylation (in all but SRC and BLK) required for membrane localization; the SH3 and SH2 domains, which are protein binding regions crucial for target interactions and also for regulating SFK catalytic activity; a linker sequence; the SH1 domain, which is the tyrosine kinase domain and contains an autophosphorylation site necessary for full activation (Tyr419 in human SRC); and the C-terminal tail, containing a negative-regulatory tyrosine residue (Tyr530 in human SRC) [25,27,55] (Figure 1A).

SFK activity is mainly regulated through phosphorylation/dephosphorylation of the conserved tyrosine residues (Tyr419 and Tyr530 in human SRC) (Figure 1B). In particular, in their inactive form, SFKs are phosphorylated in the C-terminal tail (at Tyr530 of SRC) and adopt a closed conformation through intramolecular interactions (the phosphorylated C-terminus binds the SH2 domain and the linker region binds the SH3 domain). Dephosphorylation of the negative-regulatory tyrosine causes a dramatic conformational change, resulting in autophosphorylation of the kinase domain (at Tyr419 of SRC); this auto-phosphorylation locks the catalytic domain into the active conformation and facilitates substrate access to the active site [25,27,55].

## 3. Main Roles of SFKs in Cancer

SFKs are overexpressed and hyperactivated in a wide range of cancers and contribute to several aspects of tumor development and progression, such as proliferation, survival, differentiation, migration, invasion, and angiogenesis [25,27,28,29]. As also mentioned above, one of the better-known functions of SFKs consists in their interaction (and reciprocal regulation [56]) with RTKs at the inner layer of the cell membrane and transduction of the growth and survival signals to downstream pathway effectors, such as PI3K-AKT, MAPK/ERK, and signal transducer and activator of transcription 3 (STAT3), which can also mediate angiogenesis through *VEGF* activation [25,26,27,28,29,30].

Another primary role of SFKs is to control cytoskeletal organization, cell adhesion, migration, and invasion [25,29,57,58,59], which are related processes requiring precisely orchestrated molecular interactions [25]. SFKs affect both adherens junctions and focal adhesions, the two main subcellular structures implicated in these processes [25,60,61,62,63].

Adherens junctions are multiprotein complexes, the backbone of which is composed of cadherin proteins, such as E-cadherins, which mediate cell-cell adhesion [64]. At their cytoplasmic face, E-cadherins are linked to the actin cytoskeleton through a complex consisting of α-, β-, and p120-catenins [64]. SFK signaling negatively regulates E-cadherin-mediated adhesion of cancer cells by affecting the levels, localization, and function of E-cadherins and catenins [25,62,65,66,67]. Importantly, loss of E-cadherin is a hallmark of epithelial-mesenchymal transition (EMT), which is a prerequisite for metastasis [68,69].

Focal adhesions are dynamic structures forming at the sites where integrins link the cytoskeleton to extracellular matrix (ECM) proteins and consisting of a wide variety of different components, including scaffolding proteins (e.g., p130CAS, paxillin, talin, vinculin, α-actinin) and signaling molecules, such as SRC and focal adhesion kinase (FAK) [25,70,71]. SRC can contribute to focal adhesion disassembly and cell migration, mainly through its interaction with FAK and the reciprocal full activation of these two kinases. The mutually activated FAK-SRC complex, indeed, promotes cell movement by affecting multiple downstream pathways and modulating, in particular, the activity of the RHO family of GTPases, including CDC42, RAC1, and RHOA, which coordinate the assembly of filopodia, lamellipodia, and focal adhesions, respectively [25,60,72,73].

SRC and FAK can also induce the expression and activation of the secreted matrix metalloproteinases MMP2 and MMP9 [74,75,76,77], which are responsible for the ECM remodeling required for tumor invasion. SRC is also a crucial inducer and regulator of invadopodia, which are specialized actin-rich protrusions locally degrading ECM via the activity of various proteases (membrane-type 1 (MT1)-MMP, MMP2, MMP9, ADAMs) and playing a critical role during three steps of the metastatic process: invasion into the surrounding stroma, intravasation into the vasculature, and extravasation [78,79,80].

SFK activation can promote metastasis also by contributing to resistance to anoikis, a loss of anchorage-induced type of intrinsic apoptosis [81], which would otherwise prevent detached cells from developing new tumor foci [29,82,83,84]. Moreover, SRC activation has also been implicated in the survival of disseminated cells, once they have reached distant organs, and metastatic recurrence [29].

## 4. Potential SFK Involvement in MM Development and Progression

The possible involvement of SFKs in MM development was first suggested by the observation that chickens intraperitoneally inoculated with the constitutively active *v-src* developed diffuse peritoneal MM [85]. A non-cell-autonomous role of SFKs in MM development has been also suggested by the finding that long exposure of human T cells to asbestos led to a reduction in apoptosis rate and an SFK-dependent up-regulation of the prosurvival transcription factor STAT3, implying a possible reduced antitumor immunity [86].

Analyses of both MM cell lines and tissue samples revealed that SFK hyperactivation is a common event in MM and is specific to MM cancer cells. Indeed, compared with non-tumor mesothelial cells, the active phosphorylated form of SFKs was found overexpressed in most of the well-established/commercial (MSTO-211H, NCI-H28, NCI-H2052, NCI-H2452, MPP-89, REN, ACC-MESO-4) [32,49,53,87] and primary patient-derived MM cell lines analyzed [31]. Immunohistochemical staining also showed SFK hyperactivation in MM specimens but not in normal mesothelium [31,32]. Importantly, SFK activation was found associated with a more advanced pathologic stage and the presence of metastases, thus implicating these kinases in MM progression [32].

The contribution of each SFK member to the development and progression of MM remains to be defined. Consistent with their ubiquitous expression and their role in different cancer types [27], SRC, FYN, and YES were found hyperactive and highly expressed in MM cells, suggesting their involvement in MM malignancy [31,87,88]. Moreover, the silencing of SFK members through small interfering RNAs in MM cell lines suggested a role for YES and FYN in cell growth and resistance to apoptosis, respectively [87,88]. However, further analyses through SFK genetic knock-out/modulated expression or constitutively active/inactive SFK mutants in more faithful preclinical models are necessary to dissect the exact role and possible functional redundancy of different SFK members in MM tumorigenesis and progression, assessing both cell-autonomous and non-autonomous roles, as reported for other cancer types [57,89,90,91,92]. Indeed, these studies revealed that SFK members have both unique and overlapping functions and, thus, targeting simultaneously more kinases of this family might be required to demonstrate their role. Moreover, genetically engineered mouse models proved to be particularly useful to gain insight into SFK non-cell-autonomous functions. For instance, studies in other tumors revealed that the major tumor-promoting role of LYN or HCK was mediated by microenvironmental cells, such as macrophages [90,92]. Thus, the SFK functions in MM microenvironment should be thoroughly studied in suitable preclinical models to understand fully the role of these kinases in MM progression.

## 5. Role of SFKs in Molecular Pathways Regulating Cell Adhesion, Motility, and Invasion in MM

**Figure 2:**
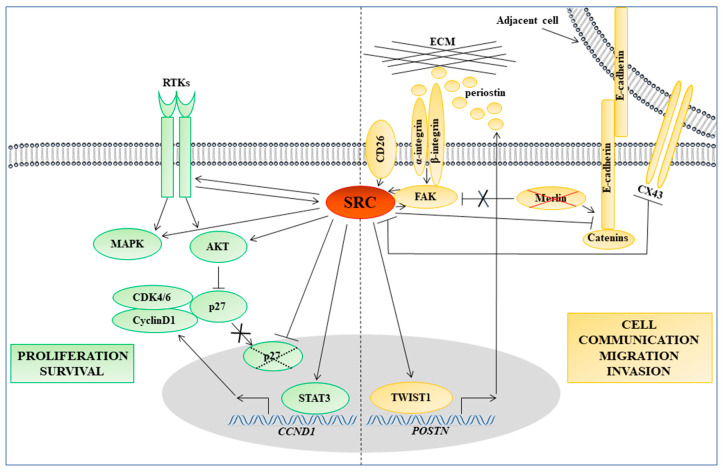
SRC-mediated molecular pathways formally assessed in malignant mesothelioma (MM). Receptor tyrosine kinases (RTKs) are frequently overexpressed and hyperactivated in MM and cooperatively interact with SRC to signal to downstream effectors, such as the mitogen-activated protein kinase (MAPK) and AKT pathways, which promote cell proliferation and survival. Moreover, SRC and AKT cooperate to inactivate the nuclear tumor suppressor activity of p27: SRC accelerates p27 proteasome-mediated proteolysis (dotted X across p27), whereas AKT inhibits p27 through various mechanisms (mentioned in the main text), including the delay in its nuclear import (black X across the arrow). SRC also induces the expression of the *CCND1* gene encoding cyclin D1, via activation of the signal transducer and activator of transcription 3 (STAT3). Cyclin D1-cyclin dependent kinase (CDK) 4/6 complex, which is a crucial component of the cell cycle machinery, can sequester p27, thus further contributing to the inhibition of its tumor-suppressive action. Different studies also implicated SRC in molecular pathways controlling MM cell communication, migration, and invasion. In particular, a crucial interacting partner of SRC is the focal adhesion kinase (FAK), a signaling component of focal adhesions, which are dynamic structures forming at the sites where integrins link the cytoskeleton to extracellular matrix (ECM) proteins. The mutually activated FAK-SRC complex promotes cell movement and invasion by affecting multiple downstream pathways. Merlin, which is a tumor suppressor protein frequently lost in MM (red X across merlin) and involved in the maturation of adherens junctions (cell-cell adhesion structures composed of cadherin and catenin proteins), negatively regulates the FAK-SRC signaling. Thus, merlin loss could be an important step in the development of the invasive properties of MM cells since it leads to the de-repression of the FAK-SRC pathway (black X across the inhibitory connector). SRC is also involved in mediating MM cell migration and invasion induced by high cell surface levels of the transmembrane glycoprotein CD26. In particular, CD26 induces the nuclear translocation of the transcription factor TWIST1 via SRC activation; this results in enhanced expression and secretion of periostin, a matricellular protein involved in the promotion of cell migration and invasion. SRC is also implicated in controlling the gap junction (GJ) intercellular communication, which occurs through the pore-forming proteins connexins (CXs) and is markedly reduced in MM cells. In particular, CX43, the most widely expressed GJ protein, is a direct substrate of SRC.

Following the identification of SFK hyperactivation in MM, various studies in vitro have revealed several molecular mechanisms whereby SFKs affect MM cell adhesion, motility, and invasion (Figure 2).

The well-known SRC target FAK, which controls cell adhesion and motility, is highly active and overexpressed in MM [93]. FAK seems to play an important role especially in MM cells lacking the *NF2* tumor suppressor gene product, merlin, which is involved in the maturation of adherens junctions [94] and is frequently lost in MM [18] (Figure 2). Indeed, merlin-deficient MM cells proved to be more sensitive to FAK inhibition, probably owing to their increased dependency on the cell-ECM-induced FAK signaling compared with merlin-positive MM cells [94]. Moreover, merlin has been identified as a negative regulator of the FAK-SRC signaling in MM: restoration of merlin expression in *NF2*-null MM cells was found to decrease significantly cell invasiveness promoted by FAK overexpression and reduce the phosphorylation of FAK at Tyr397, thus impairing its interaction with SRC and p85 (the regulatory subunit of PI3K) [95]. Considering that the binding between SRC and FAK and their reciprocal activation is fundamental to trigger downstream signaling cascades and control cell migration [60], the occurrence of this interaction as a consequence of merlin inactivation could be an important step in the development of the invasive properties of MM cells.

SRC is also involved in mediating MM cell migration and invasion induced by high cell surface levels of CD26/dipeptidyl peptidase 4 (DPP4) [46], a transmembrane glycoprotein, which has been suggested as a potential therapeutic target against MM [96]. In particular, CD26 was found to induce the nuclear translocation of the transcription factor TWIST1 via SRC activation (Figure 2); this resulted in enhanced expression and secretion of periostin [46], a matricellular protein involved in the promotion of cell migration and invasion [97], the low expression of which has been suggested as a prognostic factor for longer overall survival of MM patients [98].

A recent study also implicated SFKs in regulating the expression of MMPs in MM [45], in which these ECM-degrading enzymes associate with more invasive and aggressive behavior [99,100,101]. In particular, this study showed that activation of the G protein-coupled purinergic receptor P2Y1 by adenosine diphosphate (ADP) triggered SFK-mediated upregulation of MMP2/9 in MM cells [45]. Interestingly, this work also revealed a possible non-canonical nuclear role of MMP2/9 in the ZL55 human epithelioid MM cell line; such nuclear function of MMPs was previously identified in other tumors [102] but remains to be further addressed in MM.

Another metalloproteinase, ADAM10, was recently described as overexpressed in MM specimens (as compared with normal pleura) and contributes to MM progression by generating an N-cadherin fragment, which stimulates MM cell migration [103]. Interestingly, SRC is an ADAM10-interacting partner that positively regulates its activity in pituitary adenomas [104]; however, the role of this interaction has not yet been studied in MM.

Beyond cell adhesion, also cell-cell communication has an important role in cancer progression and metastasis [105,106]. A type of cell junction responsible for intercellular communication is the gap junction (GJ), which connects the cytoplasm of adjacent cells through the pore-forming proteins connexins (CXs) [105]. MM cell lines showed a markedly reduced GJ intercellular communication (GJIC); however, the majority of these cell lines still presented CX43 [107,108], which is the most studied and widely expressed GJ protein. CX43 is a direct substrate of SRC [109], which phosphorylates its C-terminal tail, resulting in inhibition of GJIC; moreover, there is a reciprocal regulation between CX43 and SRC, with CX43 inhibiting, in turn, SRC activity [110] (Figure 2). The role of the SRC-CX43 relationship in MM progression has not yet been thoroughly investigated, although a couple of studies found that experimentally increased levels of CX43 resulted in decreased SRC expression, reduced growth rate, and sensitization to cisplatin [47,48].

## 6. Antitumor Activity of SFK Inhibitors in MM Cell Lines and Underlying Molecular Mechanisms

Preclinical studies showed that SFK inhibitors, such as the multitargeted tyrosine kinase inhibitor dasatinib and more selective pyrazolo-[3,4-*d*]-pyrimidine SFK inhibitors (tested by our group), had antitumor effects in MM cell lines [32,49]. Dasatinib’s effects were due to both cell cycle arrest and apoptosis. Moreover, consistent with the above described role of SFKs in cell invasiveness, dasatinib also decreased MM cell migration and invasion ability [32,53]. Our pyrazolo-[3,4-*d*]-pyrimidine derivatives, which proved to be promising anticancer agents in several tumor types [44,49,111,112,113,114,115,116,117,118,119,120], had a significant antiproliferative effect selectively in MM cells with SFK hyperactivation [49]. Moreover, these molecules induced apoptosis only on cancer cells, without affecting normal mesothelial cells.

At the molecular level, both dasatinib and the pyrazolo-[3,4-*d*]-pyrimidine derivatives inhibited the activating phosphorylation of SFKs, as expected. The effects of dasatinib on the SRC targets FAK and STAT3 were also studied, revealing that this drug decreased FAK phosphorylation at the SRC target site Tyr861 and transiently reduced STAT3 activation, which returned to baseline levels upon 24 h of treatment [32]. This reactivation of the prosurvival factor STAT3 after treatment with dasatinib or other SFK inhibitors was also observed in several different cancer cell types and was suggested to be a compensatory response suppressing the antitumor effects of SFK inhibition [121]. Blocking STAT3 reactivation by combining dasatinib with Janus kinase (JAK) inhibition resulted in synergistic cytotoxicity in different tumor cells [121]; however this strategy has not yet been tested on MM cells.

Both dasatinib and our pyrazolo-[3,4-*d*]-pyrimidine derivatives decreased the active form of AKT kinase in MM cells [32,49]. This is consistent with the well-established ability of SRC to interact and activate the prosurvival factor AKT [122,123], which plays an important part in several human cancers, including MM [124,125,126,127]. A known target of both SRC and AKT is the cell cycle inhibitor p27 (Figure 2); SRC and AKT indeed cooperate to inactivate the nuclear tumor suppressor activity of p27 in human cancers: SRC phosphorylates p27 at Tyr74 and Tyr88, thus accelerating its proteasome-mediated proteolysis [128], whereas AKT delays p27 nuclear import [129], promotes its degradation [130], and inhibits its transcription [131]. Consistently, we observed that inhibitors of either SFKs or AKT induced p27 nuclear stabilization in MM cells [49,126]. Interestingly, SFK or AKT inhibitors triggered apoptosis in MM cells by mechanisms dependent, respectively, on the expression of p27 [49] and another key tumor suppressor co-regulated with p27, the RB family member RBL2/p130 [126]. However, further studies are ongoing to understand the mechanisms whereby p27 and RBL2/p130 cooperate in mediating apoptosis in MM cells upon SFK/AKT inhibition. Both dasatinib and the pyrazolo-[3,4-*d*]-pyrimidine SFK inhibitors also downregulated cyclin D1 [32,49], consistent with the role of SRC in inducing the transcription of the *CCND1* gene encoding this cyclin, via activation of the transcription factor STAT3 [132] (Figure 2). Given that cyclin D-cyclin dependent kinase (CDK)4/6 complexes, which are crucial components of the cell cycle machinery, can sequester p27 [133] and impair its cell cycle inhibitory function, the reduction in cyclin D1 could represent another mechanism whereby SFK inhibition facilitates p27 tumor-suppressive activity. Overall, these observations provide a further rationale for the use of SFK inhibitors in MM therapy. Indeed, considering that loss of nuclear p27 is a well-established adverse prognostic factor in MM [134,135,136], its expression/localization could represent a useful endpoint in SFK inhibition-based clinical trials.

## 7. SFK Inhibitors in Combination with Both Chemotherapeutics and Targeted Drugs in MM Cell Lines

Some studies performed in MM cell lines suggested that SFK inhibitors were able to increase the sensitivity to the chemotherapeutic agents currently used in MM therapy. In particular, the SFK inhibitor SU6656 enhanced cisplatin cytotoxicity in MM cells [47,48]. Moreover, dasatinib increased cisplatin-induced apoptosis of MM cells grown as multicellular spheroids, which mimic MM cell aggregates found in pleural effusions and are more resistant to cisplatin treatment [52]. Interestingly, MM spheroids expressed higher levels of phosphorylated active SFKs than cells grown in monolayer and were more sensitive to dasatinib. This suggests that SFK inhibition could serve to overcome the anoikis resistance of suspended MM cell aggregates [137], consistent with the SFK involvement in the resistance to this type of cell death observed in different tumor types [82,83,84]. However, further studies are required to understand the role of SFK activation in MM spheroid formation and resistance to anoikis and cisplatin.

Dasatinib was also able to sensitize MM cells to pemetrexed [53], which is an antimetabolite that inhibits different folate-dependent enzymes, such as thymidylate synthase (TS). The limited efficacy of pemetrexed against MM could be due to resistance mechanisms, including high TS levels. SRC was found to control the expression of this enzyme at the transcriptional level and, consistently, dasatinib suppressed the pemetrexed-induced up-regulation of TS [53]. Thus, dasatinib seemed to improve MM cell sensitivity to pemetrexed by down-regulating TS.

Beyond sensitizing MM cells to the currently used chemotherapeutics, SFK inhibitors could also be useful in combination with inhibitors of other tyrosine kinases, the signaling of which is aberrantly activated in MM. Indeed, tyrosine kinases such as EGFR, MET, and SFKs can redundantly signal to downstream oncogenic pathways, including PI3K-AKT-mTOR and MAPK (Figure 2); thus, inhibition of one of these tyrosine kinases alone in MM cells might be ineffective if the other kinases induce the same cell proliferation/survival pathways [31]. Consistently, the SFK inhibitor PP2 in combination with either MET or EGFR inhibition was more effective than each tyrosine kinase inhibitor alone in reducing cell viability of MM cell lines. Moreover, combined tyrosine kinase inhibitors were more efficient than a tyrosine kinase inhibitor alone in reducing phosphorylation of AKT, ERK1/2, and S6 ribosomal protein, which is indicative of inactivation of the downstream pathways PI3K-AKT, MAPK, and mTOR, respectively. In particular, the EGFR-SFK inhibitor combination was the most effective treatment [31]. The optimization of such combination strategies could help to avoid and/or tackle the emergence of resistance associated with these agents.

SFK inhibitors also proved to enhance the antitumor effects of immunotoxins against mesothelin, which is a cell-surface glycoprotein overexpressed in MM and other cancers; however, the combination of these agents has not yet been tested on MM cells [138]. Also, SFK inhibition has been implicated in the mechanism of action of different agents with antitumor activity in MM cells [47,48,139,140], thus further supporting the importance of inactivating these kinases in treatments against MM.

Overall, the preclinical studies described above suggest a potential application of SFK inhibition, both alone and in combination with other treatments, in MM therapy. However, these preclinical data should be interpreted with caution, taking into account that the SFK inhibitors used in these studies target also other kinases (Table 1), with similar or even greater potency [43]. For instance, the compound SU6656 was found to inhibit Aurora B and C kinases, more potently than SFKs in vitro [43]. Therefore, further studies are necessary to dissect the contribution of single kinase inhibition to the overall anticancer effects.

## 8. Clinical Trials of Dasatinib in MM Patients

Although the abovementioned preclinical studies pointed to SFK inhibitors as promising therapeutic agents for MM, unfortunately, two recent clinical trials using dasatinib in unselected MM patients did not show efficacy [50,51]. Dasatinib is an orally administered drug used in the treatment of chronic myeloid leukemia and Philadelphia chromosome-positive acute lymphoblastic leukemia. This drug inhibits multiple tyrosine kinases, as revealed by in vitro kinase activity assays. In particular, dasatinib potently inhibits SFKs, BCR-ABL fusion protein, and CSF-1R, with half maximal inhibitory concentration (IC50) values < 1 nM, and also has significant activity against KIT, EPHA2, and PDGFRB, although with IC50 values approximately 10, 30, and 60 fold higher than for SFKs, respectively [33,34,35]. Moreover, dasatinib inhibits also other kinases, but with much lower potency (IC50 values ranging from 100 nM to >50 µM) [33]. Dasatinib showed a broad target profile also through affinity-based proteomic strategies, which allow the parallel determination of protein-binding profiles of kinase inhibitors in any cell type under more physiological conditions compared with in vitro kinase activity assays [141,142,143].

A phase II study evaluated dasatinib as a second-line treatment in 43 patients with unresectable MM no longer controlled with first-line platinum and pemetrexed therapy [50]. The most common grade 3 and 4 adverse events were fatigue (11%) and pleural effusion (9%). The overall disease control rate was 32.6% and progression-free survival (PFS) at 24 weeks was 23%, which is below the prespecified 42% needed to declare the regimen worthy of further study. Considering that SRC acts downstream the CSF-1 pathway [144,145] and given that increased serum levels of this cytokine associate with higher aggressiveness of different tumor types [50], this study also analyzed the serum levels of CSF-1 in MM patients. Interestingly, overall survival was markedly longer in patients with lower serum levels of CSF-1 before treatment and PFS was approximately three times longer in patients whose CSF-1 levels decreased from baseline during therapy.

A second clinical study evaluated dasatinib as a neoadjuvant agent in 24 patients with resectable MM [51]. In particular, in this window of opportunity trial, patients were treated for 4 weeks with oral dasatinib until the day before they underwent surgery with or without radiotherapy. The main side effects were grade 1 to 2 fatigue, anorexia, nausea, and edema. No significant responses were seen after this short period of dasatinib therapy; however, median immunohistochemistry scores for active phosphorylated SFKs significantly decreased after treatment and the change in SFK activation status correlated with radiographic response by standardized uptake value (SUV) levels on positron emission tomography/computed tomography (PET/CT) scan. In particular, patients with decreased SFK activation were more likely to have a decrease in SUV levels, whereas those with increased SFK activation were more likely to have an increase in SUV levels. Also, patients with reduced scores for active phosphorylated SFKs had a longer median PFS than patients with increased scores. Interestingly, two patients who received maintenance adjuvant dasatinib remained disease-free during the treatment (18.5 and 31.5 months) but MM progressed upon dasatinib therapy cessation. This suggests that adjuvant consolidation dasatinib could improve PFS, although further studies are obviously required. Importantly, higher average baseline membrane levels of active phosphorylated SFKs were predictive for a decrease in SUV levels after neoadjuvant dasatinib. Moreover, distinct PDGFR biomarker phenotypes at baseline had predictive value for dasatinib. In particular, patients with higher PDGFRB levels had a worse PFS, thus suggesting that high expression of PDGFRB could be a mechanism of resistance to dasatinib.

## 9. Conclusions and Future Directions

MM is a lethal asbestos-induced cancer for which there is currently no curative modality, although several promising therapeutic targets have been identified over the years. Unfortunately, however, most strategies, which were successful against MM in preclinical studies, failed when attempted in the clinical setting. Many hurdles have hampered a successful translation of basic discoveries. First, most preclinical models are quite limited and unable to represent the enormous complexity of the MM milieu [146]. MM cells with peculiar genetic and epigenetic features develop and progress interacting initially within a microenvironment, mostly through auto and paracrine signaling within neighboring tissues, while at later stages the tumor-induced angiogenesis and lymphatic spreads increase the range of systemic interactions within a more complex macroenvironment. Therefore, it is extremely urgent to implement MM research with preclinical models better suited to recapitulate these features to gain a more thorough understanding of both the cell-autonomous and non-cell-autonomous molecular mechanisms underlying MM tumorigenesis.

Another key hurdle in successfully translating basic discoveries into effective new therapies against this cancer is that, although many possible molecular targets have been identified, there are few reliable biomarkers and, therefore, clinical trials are generally performed on unselected patient cohorts [14]. However, MM, despite having a predominant etiology, linked to asbestos exposure, is highly molecularly heterogeneous, as recently evidenced through genomic profiling studies [7,11,13,147], and more personalized approaches are required to improve clinical results. To this purpose, it is crucial to comprehensively identify druggable molecular vulnerabilities in subsets of MMs, also finding relevant biomarkers or predictive signatures that allow a more tailored patients’ selection [11]. Considering that MM is a rare disease, large randomized clinical trials with selected patients are difficult to realize; the need to operate in consortium and through standardized methods has been also recently emphasized [148].

SFKs are particularly appealing targets for anticancer therapies because they are aberrantly activated in most tumor types and their inhibition can affect multiple signaling pathways implicated in proliferation, survival, differentiation, migration, invasion, metastasis, and angiogenesis [25,27,28,29]. Considering that SFK hyperactivation frequently occurs in MM [31,32], in which it associates with advanced and metastatic stages [32] and contributes to the alteration of many molecular pathways (Figure 2), SFKs represent promising therapeutic targets also against this cancer. Consistently, preclinical studies by our group and others showed that SFK inhibitors had antiproliferative effects and caused a decrease in migration and invasion in MM cell lines [32,49]. Moreover, SFK inhibitors enhanced the sensitivity of MM cells to the chemotherapic agents (cisplatin and pemetrexed) currently used in MM therapy [47,48,52,53]. However, clinical trials using dasatinib both as a second-line treatment for patients with unresectable MM [50] and as a neoadjuvant agent in patients with resectable MM [51] did not show efficacy.

These results are in line with what observed for many other solid tumors. Indeed, although extensive preclinical evidence suggested that targeting SFKs could be an effective anticancer strategy for several tumor types, results from clinical trials of SFK inhibitors were not encouraging [29,149]. One likely reason for these disappointing outcomes is that most clinical studies of SFK inhibitors, including the trials for MM, involved unselected patients. Preclinical studies pointed to the critical importance of SFK hyperactivation for the therapeutic efficacy of SFK inhibitors; however simple measurement of the expression of the activated form of SFKs is unlikely to predict the response to their inhibitors since other molecular alterations can have an impact [29,150]. Some studies defined gene expression profiles that can predict sensitivity to SFK inhibitors in cell lines from different solid tumors. In particular, a study identified a gene expression signature mirroring the activation status of the SRC pathway, which can predict the sensitivity of a broad range of tumor cell lines to the SFK inhibitor SU6656 [151]. Other studies identified sets of genes correlated with the sensitivity of cell lines from breast, lung, prostate, and ovarian cancers to dasatinib [34,152,153,154]. Interestingly, some of the genes related to dasatinib sensitivity were commonly identified in independent studies on different cell types. Moreover, markers for response prediction to dasatinib were also found in uterine cancer cells, by using reverse-phase protein array [155], and in lung cancer cell lines, through quantitative mass spectrometry to profile the phosphoproteome [156]. Overall, many of the identified factors were targets of dasatinib, substrates of SFKs, and components of signaling pathways downstream of SFKs involved in cell adhesion, cytoskeleton organization, and migration. Definition of similar predictive profiles in MM cells could serve to guide the design of future clinical trials of SKF inhibitors.

Importantly, the large amount of knowledge on SFKs and their interacting partners supports the view that rational combinations of SFK inhibitors with other molecularly targeted therapeutics could potentially increase the clinical benefit with manageable toxic effects [29,150]. In particular, different preclinical studies showed that targeting SFKs enhanced the efficacy of anti-RTK drugs in cells from different tumor types [29]. SFKs are, indeed, involved in multiple resistance mechanisms to anti-RTK therapies; moreover, RTKs are, in turn, implicated in resistance to SFK inhibitors [29]. In MM cell lines, as well as in other cancer cell types, SFKs and RTKs, such as EGFR and MET, are often concomitantly activated and can positively regulate each other and promote cell survival and resistance to single tyrosine kinase inhibition, by redundantly signaling to the same downstream pathways, including PI3K-AKT-mTOR and MAPK [31]. Consistently, as described above, the SFK inhibitor PP2 in combination with either MET or EGFR inhibition was more effective than each tyrosine kinase inhibitor alone in reducing MM cell viability [31]. These observations support the hypothesis that tyrosine kinase inhibitor monotherapies could be ineffective against MM, in line with the data from clinical studies exploring, for example, the effects of EGFR inhibition in MM patients [13]. Moreover, the results from both the clinical trials of dasatinib in MM patients further support the notion that the RTK signaling is involved in resistance to SFK inhibition. In particular, in these trials, the baseline levels of CSF-1 [50] and PDGFR [51] seemed to affect the response to dasatinib.

Also, it will be interesting to evaluate the possible combination of SFK inhibitors with antioangiogenic drugs. Targeting angiogenesis has been a highly pursued strategy against MM and, in particular, blocking VEGF through bevacizumab in addition to the pemetrexed-platinum doublet improved PFS and overall survival, as assessed in the first large Phase III MAPS trial, spurring enthusiasm in the field [157,158]. Preclinical studies in other tumors suggest that the concomitant inhibition of VEGF signalling and SFKs potentiates antitumoral effects by stabilizing the endothelial barrier function, thus preventing tumor cell extravasation, and overcoming drug resistance [159,160,161,162]. It will be worth exploring whether also in MM bevacizumab and SFK inhibition can tackle disease progression.

Finally, for a successful use of SFK inhibition as anticancer therapy, not only the tumor cell-specific but also the non-cell-autonomous roles of SFKs will have to be considered. Indeed, SFKs have been defined as rheostats of immune cell signaling [163] and, therefore, the impact of their pharmacological inhibition on players of the immune response will have to be carefully dissected. In head and neck squamous cell carcinoma, dasatinib acted synergistically with cytotoxic T-lymphocyte associated protein 4 (CTLA4) blockade, suggesting yet another avenue of investigation [164]. As mentioned above, specific SFK members also play a crucial part in regulating the macrophage-promoted progression of different cancer types [90,92]. Interestingly, SFKs are crucial mediators of the CSF-1-CSF-1R-induced maturation of migratory tumor-associated macrophages [165]. These microenvironmental cells contribute to tumor growth, angiogenesis, invasion, metastasis, immunosuppression, and resistance to anticancer therapies; therefore, CSF-1/CSF1-R inhibitors, both alone and in combination with chemotherapy, radiotherapy, and immunotherapies, are in clinical development for several tumor types, including MM [166,167]. Thus, these observations point to the importance of thoroughly investigating the possible role of SFKs in MM-associated macrophages. Beyond immune cell players, SFKs likely affect also other components of the microenvironment; studies in Drosophila show that the SRC homologue contributes to non-autonomous tumorigenesis acting within an oncogenic niche stimulating neighbor tissue overgrowth [168]. This work further emphasizes that studying the complex tumor topography is crucial to conceive and apply precision medicine strategies that affect a whole set of different cell types and interactions.

In conclusion, although SFKs are promising therapeutic targets for MM, the complexity of their signaling pathways hinders the successful translation of SFK inhibitors into the clinic. The definition of reliable predictive markers/signatures for selection of MM patients who are most likely to benefit from SFK inhibition-based therapy, a more thorough assessment of the effect of SFK inhibition on the complex micro and macroenvironment in more faithful preclinical models, and the design of rational combinatorial regimens will likely contribute to a more successful application of these agents in MM therapy.

## Figures and Tables

**Table 1:** Selected drugs targeting SFKs and other kinases.

Drug	Main Targets	Status	References
**DASATINIB** * (BMS354825) *N*-(2-chloro-6-methylphenyl)-2-[[6-[4-(2-hydroxyethyl)piperazin-1-yl]-2-methylpyrimidin-4-yl]amino]-1,3-thiazole-5-carboxamide	BCR-ABL, CSF-1R, EPHA2, KIT, PDGFRB, SFKs	Clinical trials/FDA approved for CML and Ph+ ALL	[33,34,35,50,51]
**BOSUTINIB** (SKI-606) 4-[(2,4-Dichloro-5-methoxyphenyl)amino]-6-methoxy-7-[3-(4-methyl-1-piperazinyl)propoxy]-3-quinolinecarbonitrile	BCR-ABL, SFKs	Clinical trials/FDA approved for CML	[36]
**PONATINIB** (AP24534) 3-(imidazo [1,2-b]pyridazin3-ylethynyl)-4-methyl-*N*-19benzamide hydrochloride benzamide	BCR-ABL, KIT, FGFR1, FLT1, PDGFR, RET, SFKs	Clinical trials/FDA approved for CML and Ph+ ALL	[37]
**SARACATINIB** (AZD0530) *N*-(5-chloro-1,3-benzodioxol-4-yl)-7- [2-(4-methylpiperazin-1-yl)ethoxy]-5-(oxan-4-yloxy) quinazolin-4-amine	BCR-ABL, SFKs	Clinical trials	[38]
**AZD0424** 1-[4-[2-[4-[(6-chloro-[1,3]dioxolo [4,5-b]pyridin-7-yl)amino]-5-propan-2-yloxyquinazolin-7-yl]oxyethyl]piperazin-1-yl]ethanone	BCR-ABL, SFKs	Clinical trials	[39]
**KXO1**^†^ (KX2-391) *N*-benzyl-2-[5-[4-(2-morpholin-4-ylethoxy)phenyl]pyridin-2-yl]acetamide	SFKs, Tubulin	Clinical trials	[40]
**PP1** * 1-*tert*-butyl-3-(4-methylphenyl)pyrazolo [3,4*d*] pyrimidin-4-amine	EPHA2, FGFR1, KIT, MAPK, RIP2, TGF-β type I, SFKs	Preclinical studies	[41,42,43,45]
**PP2** * 1-*tert*-butyl-3-(4-chlorophenyl)pyrazolo [3,4-*d*]pyrimidin-4-amine	CK1δ, EPHA2, FGFR1, KIT, MAPK, RIP2,TGF-β type I, SFKs	Preclinical studies	[41,42,43,46]
**SU6656** * (3*Z*)-*N*,*N*-dimethyl-2-oxo-3-(4,5,6,7-tetrahydro-1*H*-indol-2-ylmethylidene)-1*H*-indole-5-sulfonamide	AMPK, AURORA B/C, BRSK2, MST2, SFKs	Preclinical studies	[43,47,48]
**SI83** * and **SI91** * pyrazolo [3,4-*d*]pyrimidines derivatives	BCR-ABL, SFKs	Preclinical studies	[44,49]

The table reports the main SFK inhibitors used in clinical trials for different cancer types. In addition, SFK inhibitors used in preclinical studies in malignant mesothelioma (MM) cells are also included. * Drugs that were used in MM at either the preclinical or clinical level. ^†^ KXO1 is a non-ATP-competitive drug, whereas all the other drugs reported are ATP-competitive. Abbreviations: AMPK, AMP-activated protein kinase; BRSK2, BR serine/threonine kinase 2; CK1δ, casein kinase 1δ, CML, chronic myeloid leukemia; CSF-1R, colony-stimulating factor 1 receptor; FDA, Food and Drug Administration; FGFR1, fibroblast growth factor receptor 1; FLT1, fms related receptor tyrosine kinase 1; MAPK, mitogen-activated protein kinase; MST2, mammalian STE20-like kinase 2; PDGFR, platelet-derived growth factor receptor; Ph+ ALL, Philadelphia chromosome–positive acute lymphoblastic leukemia; RIP2, receptor interacting serine/threonine kinase 2; SFKs, SRC family kinases; TGF-β type I, transforming growth factor β receptor type I.
